# Super‐enhancer‐driven KIAA1522 upregulation suppresses ferroptosis in hepatocellular carcinoma

**DOI:** 10.1002/ctm2.70710

**Published:** 2026-06-11

**Authors:** Xinyang Li, Siyuan Hu, Haolin Shi, Pan Bian, Ziming Wang, Jiachun Sun

**Affiliations:** ^1^ Henan Key Laboratory of Cancer Epigenetics Cancer Institute The First Affiliated Hospital College of Clinical Medicine Henan University of Science and Technology Luoyang China

**Keywords:** ferroptosis, hepatocellular carcinoma, KIAA1522, super‐enhancers

## Abstract

**Background:**

Aberrantly activated super‐enhancers (SEs) drive hepatocellular carcinoma (HCC) malignancy. However, the sustained oncogenic mechanisms mediated by SE‐driven genes remain elusive.

**Methods:**

HCC‐specific SE genes were identified by cross‐analysing H3K27ac ChIP‐seq and RNA sequencing (RNA‐seq). Analyses of HCC databases using Cox proportional hazards regression were performed to identify core SE‐activated genes. The SE‐mediated transcriptional activation of an identified gene (i.e., KIAA1522) was validated through SE landscapes, ChIP‑qPCR, CRISPR interference and dual‑luciferase reporter assays. HCC cells with knockdown or overexpression of KIAA1522 were constructed to evaluate its functional impact, and RNA‐seq was performed to identify downstream pathways. Ferroptosis was evaluated by measuring the levels of malondialdehyde (MDA), reactive oxygen species (ROS) and ferrous ions.

**Results:**

SE inhibitors suppressed HCC cell proliferation. KIAA1522 was identified as an HCC‐specific SE gene, exhibiting elevated expression in HCC tissues and an association with unfavourable outcomes. Targeting SEs reduced KIAA1522 expression, and the SE region at chr1: 32753965‒32762956 transcriptionally activated KIAA1522. Functionally, KIAA1522 knockdown exerted anti‐proliferative effects in vitro and in vivo. Furthermore, its knockdown triggered ferroptosis, elevated the levels of MDA, ROS and ferrous ions, and downregulated the expression of hallmark proteins. Conversely, KIAA1522 overexpression reversed these effects. Notably, targeting the SEs recapitulated the phenotypic consequences of KIAA1522 knockdown. Mechanistically, activating transcription factor 4 (ATF4) serves as a key downstream effector of KIAA1522, playing an essential role in ferroptosis triggered by KIAA1522 knockdown.

**Conclusion:**

As a SE‐driven oncogene, KIAA1522 facilitates HCC progression via evasion of ferroptosis, representing an actionable target with potential for clinical translation.

**Key points:**

- Aberrantly activated SEs drive the overexpression of the oncogene KIAA1522 in HCC.
- Targeting SEs and knocking down KIAA1522 facilitate ferroptosis in HCC.
- As a key downstream effector of KIAA1522, ATF4 plays an essential regulatory role in ferroptosis mediated by the oncoprotein.

## Graphical abstract

Critical points:

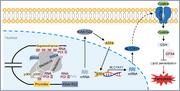

## 1 INTRODUCTION

Hepatocellular carcinoma (HCC) poses a formidable challenge to clinical practice.^1^ Early diagnosis of HCC is rare, with more than half of patients presenting at an advanced stage of the disease, thus precluding curative treatment.^2^ Currently available options for systemic therapy, including targeted and immunotherapeutic approaches, have enhanced survival for a proportion of the patients with HCC. However, clinical response rates remains modest, and there is significant room for improvement in long‐term prognosis.^3^ Therefore, there is a critical need for further investigation into the pathogenesis of HCC.

Tumour initiation and progression are driven by genetic and epigenetic aberrations.^4^, ^5^ Epigenetic mechanisms regulate gene expression and cell fate without altering DNA, facilitating malignant growth, immune evasion and resistance to therapy by modifying the structure of chromatin.^6^ Aberrant epigenetic regulation can lead to alteration of the gene transcription initiation programs.^7^, ^8^ The role of super‐enhancers (SEs) in this process has garnered significant attention owing to their exceptionally strong capacity for transcriptional activation.^9^ SEs represent potent cis‐regulatory regions enriched for H3K27ac and H3K4me1.^10^ They efficiently recruit transcriptional coactivators and interact with promoters to drive high‐level gene expression, which is crucial for cell identity.^11^ Studies have shown that SEs are abnormally activated in various malignant tumours and mediate signalling pathway dysregulation and abnormal cell proliferation, thereby becoming key oncogenic drivers.^12^

The concept of SEs originated from research on acute T‐lymphoblastic leukemia; the evidence revealed that SEs potently drive oncogene expression, thereby establishing their role in transcriptional regulation.^13^ Currently, the role of SEs in various solid tumours has been widely confirmed. In colon cancer, SEs regulate the activity of the wingless‐related integration site (Wnt) signalling pathway through enriching Wnt‐related transcription factors.^14^ In lung cancer, they are involved in mediating resistance to chemotherapy.^15^ In breast cancer, the heterogeneous landscape of SEs can serve as an effective tool for internal subtyping, capable of identifying high‐risk subtypes with distinct poor prognosis and specific therapeutic responses.^16^ Furthermore, SEs have been confirmed to be closely associated with malignant processes, such as tumour epithelial‒mesenchymal transition, apoptosis resistance and metabolic reprogramming.^17^ Notably, therapeutic strategies targeting SEs have shown promising application prospects. Agents such as SEs inhibitors (JQ‐1 and i‐BET151) and transcriptional kinase inhibitors can effectively suppress the SE‐driven oncogenic transcription program in various tumour models, providing a new direction for precision cancer therapy.^18^ In recent years, SEs have been identified as pivotal regulators of HCC pathogenesis.^19^ Their dysregulation is implicated in HCC aggressiveness; hence, exploring the potential usefulness of SEs in targeted therapies for HCC is an emerging research direction.^20^

The objective of this study was to systematically decipher the transcriptional regulatory network of SEs in HCC by integrating multi‐omics data and functional experiments. We found that SE inhibitors significantly inhibited HCC cell proliferation. Through H3K27ac chromatin immunoprecipitation‐sequencing (ChIP‐seq), ChIP‐quantitative polymerase chain reaction (ChIP‐qPCR) and dual‐luciferase reporter assays, we identified an HCC‐specific SE target gene (i.e., KIAA1522), which is aberrantly activated by a SE region. Functionally, KIAA1522 knockdown suppressed cell proliferation and tumour growth in vivo; moreover, it triggered ferroptosis, manifested as enhanced lipid peroxidation, disrupted antioxidant systems and abnormal mitochondrial morphology. Notably, targeting the SEs recapitulated the phenotypic consequences of KIAA1522 knockdown. Conversely, KIAA1522 overexpression produced the opposite phenotype. Clinical analysis validated that elevated levels of KIAA1522 predict unfavourable outcomes in HCC. Mechanistically, KIAA1522 confers resistance to ferroptosis by regulating activating transcription factor 4 (ATF4) and solute carrier family 7 member 11 (SLC7A11). The findings of this study establish the pivotal function of the SEs/KIAA1522/ferroptosis cascade in HCC, identifying a promising intervention target for this malignancy.

## 2 METHODS

### 2.1 Cell culture

HCC cell lines (HuH7, HepG2, MHCC97H, Hep3B and HCC‐LM3) were purchased from Wuhan Servicebio; THLE‐2 and HEK‐293T were purchased from Wuhan Procell. Cells were authenticated by short tandem repeat profiling and cultured according to instructions.

### 2.2 Cell transfection

The PLKO.1‐puro lentiviral vector (Addgene) contained shRNAs against KIAA1522 and bromodomain‐containing protein 4 (BRD4) prepared by JTSBIO. Simultaneously, the PLVX‐puro lentiviral vector harboured the custom sequences encoding KIAA1522 (NM_020888.3) and ATF4 (NM_182810.3) to generate KIAA1522 and ATF4 overexpression plasmids. Additionally, the pLVhU6‐sgRNA hUbC‐dCas9‐KRAB‐T2a‐puro vector (Addgene) harboured designed CRISPR guide RNAs directed against the SE genome. Calcium phosphate transfection kit (Beyotime) was employed to introduce pMD2.G, psPAX2 and target plasmids (all from Addgene) into HEK‐293T cells. The primers are listed in Table S1.

### 2.3 Cell viability assay

Prior to treatment, HCC cells were seeded into 96‐well plates (5 × 10^3^ cells/well) by group. Subsequently, drug interventions were performed with JQ‐1 (1.25 and 2.5 µM, MCE) or i‐BET151 (.5 and 1 µM, MCE). For phenotypic studies following genetic interventions, cells with knockdown or overexpression were replated into 96‐well plates. Cell viability was quantified by cell counting kit‐8 (CCK‐8) assay (Beyotime).

### 2.4 5‑Ethynyl‑2′‑deoxyuridine assay

Following 48 h of drug (JQ‐1: 1.25 and 2.5 µM; i‐BET151: .5 and 1 µM) or genetic (knockdown/overexpression) intervention in six‐well plates (1 × 10^6^ cells/well), HCC cells were exposed to 10 µM 5‑ethynyl‑2′‑deoxyuridine (EdU) working solution (Beyotime). Subsequently, 4% paraformaldehyde (Seven) and 4% permeabilisation buffer (Beyotime) were applied for cell fixation and permeabilisation, followed by exposure to click reaction mixture and mounting with DAPI‐containing medium (Beyotime). Final fluorescence images were quantified via ImageJ (1.54 g).

### 2.5 Colony formation assay

Following 2 weeks of treatment with JQ‐1 (1.25 and 2.5 µM) or i‐BET151 (.5 and 1 µM) in six‐well plates (3 × 10^3^ cells/well), HCC cells were processed for fixation and staining. Subsequently, image acquisition was performed and data were quantified via ImageJ.

### 2.6 Cell apoptosis assay

Following treatment with JQ‐1 (1.25 and 2.5 µM) or i‐BET151 (.5 and 1 µM) for 48 h, HCC cells were harvested and placed in buffer. Next, cells were stained with annexin V‐fluorescein isothiocyanate (FITC) and propidium iodide (PI) from an apoptosis detection kit (Beyotime), and analysed via CytoFLEX LX flow cytometry (Beckman).

### 2.7 Characterisation of SEs and effector genes

Total RNA derived from Huh‐7 and HepG2 cells exposed to 2.5 µM JQ‐1 for 48 h was purified with TRIzol reagent (Invitrogen) and subjected to RNA sequencing (RNA‐seq) on DNBSEQ‐T7RS (MGI Tech). Differentially expressed genes (DEGs) between cells with and without JQ‐1 were recognised in R Studio (4.4.2) with |Log_2_ FC| ≥ 1 and adj. *p* < .05. Subsequently, these DEGs were intersected with HCC‐specific SE genes from the SEdb 3.0^21^ to identify downregulated, HCC‐specific SE‐linked genes. Furthermore, HCC clinical patient data and transcriptomic sequencing data were sourced from The Cancer Genome Atlas (TCGA) and the Gene Expression Omnibus. Potential SE‐associated genes with independent prognostic value were identified via COX regression analysis. Prognostic evaluation for overall survival (OS), recurrence‐free survival (RFS), progression‐free survival (PFS) and disease‐specific survival (DSS) was accomplished using data from the TCGA and Kaplan‒Meier Plotter databases. Additionally, H3K27ac, H3K4me1, H3K4me3 and BRD4 ChIP‐seq profiles in Huh7 and HepG2 cells were retrieved from Cistrome DB and visualised with UCSC genome browser.

### 2.8 Quantitative reverse transcription PCR

Total RNA from cells and tissues was purified and converted into complementary DNA with RNA extraction (Solarbio) and reverse transcription kits (Yeasen), followed by quantitative reverse transcription PCR (qRT‐PCR) with SYBR (Yeasen) on an ABI 7500 System (Invitrogen). The primers are listed in Table S2.

### 2.9 Western blotting

Protein harvested with RIPA (Beyotime) was resolved by electrophoresis and electrotransferred onto membranes (Millipore). Subsequently, the membranes were probed with primary and corresponding secondary antibodies, followed by detection with ECL reagent (Seven) via the JiaPeng imaging system. Finally, the intensities of protein bands were determined using ImageJ. The antibody details are provided in Table S3.

### 2.10 Clinical HCC samples

In this study, 60 paired HCC and peritumoural normal tissue specimens and 15 fresh matched samples for KIAA1522 expression profiling were used. The study was approved by the ethics committee of the First Affiliated Hospital of Henan University of Science and Technology (K‐2025‐B013).

### 2.11 Immunohistochemistry

Sections were subjected to epitope retrieval and quenching, followed by probing with an anti‐KIAA1522 antibody (1:100, Invitrogen), DAB visualisation and nuclear counterstaining. Quantitation was conducted using the immunohistochemistry (IHC) Profiler plugin in ImageJ. The H‐score (0‒300) was calculated as follows: 3 × (percentage of high positive cells) + 2 × (percentage of positive cells) + (percentage of low positive cells).

### 2.12 Wound‑healing and Transwell invasion assay

For migration assessment, wounds were generated with a 200 µL pipette tip in approximately 90% confluent HCC cell monolayers and photographed at 0 and 24 h to determine closure. For invasion assay, pretreated serum‐deprived HCC cells were loaded in the upper compartment of Matrigel‐covered Transwell inserts (Corning), while the lower compartment contained medium supplemented with 30% foetal bovine serum. Transmigrated cells were quantified with ImageJ after 48 h.

### 2.13 ChIP‒qPCR assay

Chromatin from HCC cells was pulled down by anti‐BRD4 antibody (CST) employing the SimpleChIP Plus Enzymatic Chromatin IP Kit (CST). Immunoprecipitated DNA was quantified by qPCR with QuantityNova SYBR Green PCR (Qiagen). The primers are listed in Table S2.

### 2.14 Dual‑luciferase reporter assay

The KIAA1522 promoter and SE fragments were assembled, inserted into pGL3.0‐basic (Tsingke), and co‐transfected into cells alongside pRL‐TK (Renilla) for normalisation. After 24 h, dual‐luciferase activity was determined using an assay kit (Meilunbio) on a Multi‐Mode Detection Platform (Molecular Devices). The relevant DNA sequences are listed in Table S1.

The putative ATF4‐binding element within the SLC7A11 promoter was retrieved from JASPAR. Based on a previous study performed by Ji et al.,^22^ the wild‐type and mutant promoter fragments were subcloned into pGL3.0‐basic, and co‐transfected with pRL‐TK (Renilla) into ATF4‐overexpressing or empty vector control HEK‐293T cells. At 48 h post‐transfection, luciferase activity was quantified via Multi‐Mode Detection Platform.

### 2.15 Tumourigenesis assay in vivo

This animal study was approved by the experimental animal ethics committee of the First Affiliated Hospital of Henan University of Science and Technology (D‐2025‐B012). Male BALB/C nude mice (6 weeks old) used in the experiment were purchased from Huafukang Bioscience. All mice were maintained under specific pathogen‐free conditions (23°C, 12‐h light/dark cycle), with ad libitum access to water and food.

To establish the xenograft tumour model, HepG2 cells stably expressing either control (shNC) or KIAA1522‐knockdown (shKIAA1522‐1 and shKIAA1522‐2) were subcutaneously implanted into the backs of mice (5 × 10^6^ cells/animal). Once tumours became palpable, mice were randomly assigned to the tumour growth group (*n* = 6/group) or survival cohorts (*n* = 12/group). Tumour dimensions were measured every 3 days, and volume (mm^3^) was determined as (length × width^2^) × .5. Finally, xenograft tumours were harvested, weighed and stored following euthanasia.

### 2.16 Mechanism exploration

RNA‐seq was performed in KIAA1522‐knockdown HepG2 cells via the DNBSEQ‐T7RS platform, and Gene Set Enrichment Analysis (GSEA) was employed to decipher putative signalling cascades.

### 2.17 GSH/GSSG, MDA, ferrous ion and ROS assay

For the characterisation of ferroptosis, stably KIAA1522‐knockdown cells (Huh7 and HepG2) were incubated with 10 µM Erastin (MCE) or 2 µM Ferrostatin‐1 (Fer‐1, MCE) for 24 h; KIAA1522‐overexpressing HCC‐LM3 cells were cultured without drug intervention. Next, the cells were harvested for glutathione/glutathione disulfide (GSH/GSSG) (reduced/oxidised glutathione), malondialdehyde (MDA) ferrous ions and reactive oxygen species (ROS) levels via Beyotime kits; ROS levels were quantified using a flow cytometer (Beckman).

### 2.18 Transmission electron microscopy

Stably KIAA1522‐knockdown Huh7 and HepG2 cells were fixed, dehydrated, sectioned and photographed under a transmission electron microscope (Hitachi).

### 2.19 Statistical analysis

GraphPad Prism (10.1.2) was applied for statistical analysis. Data are presented as the mean ± SD. Student's *t*‐test and one‐way analysis of variance (ANOVA) for normally distributed data. *p* < .05 defined statistical significance.

## 3 RESULTS

### 3.1 Targeting SEs suppresses HCC cell proliferation

**FIGURE 1:**
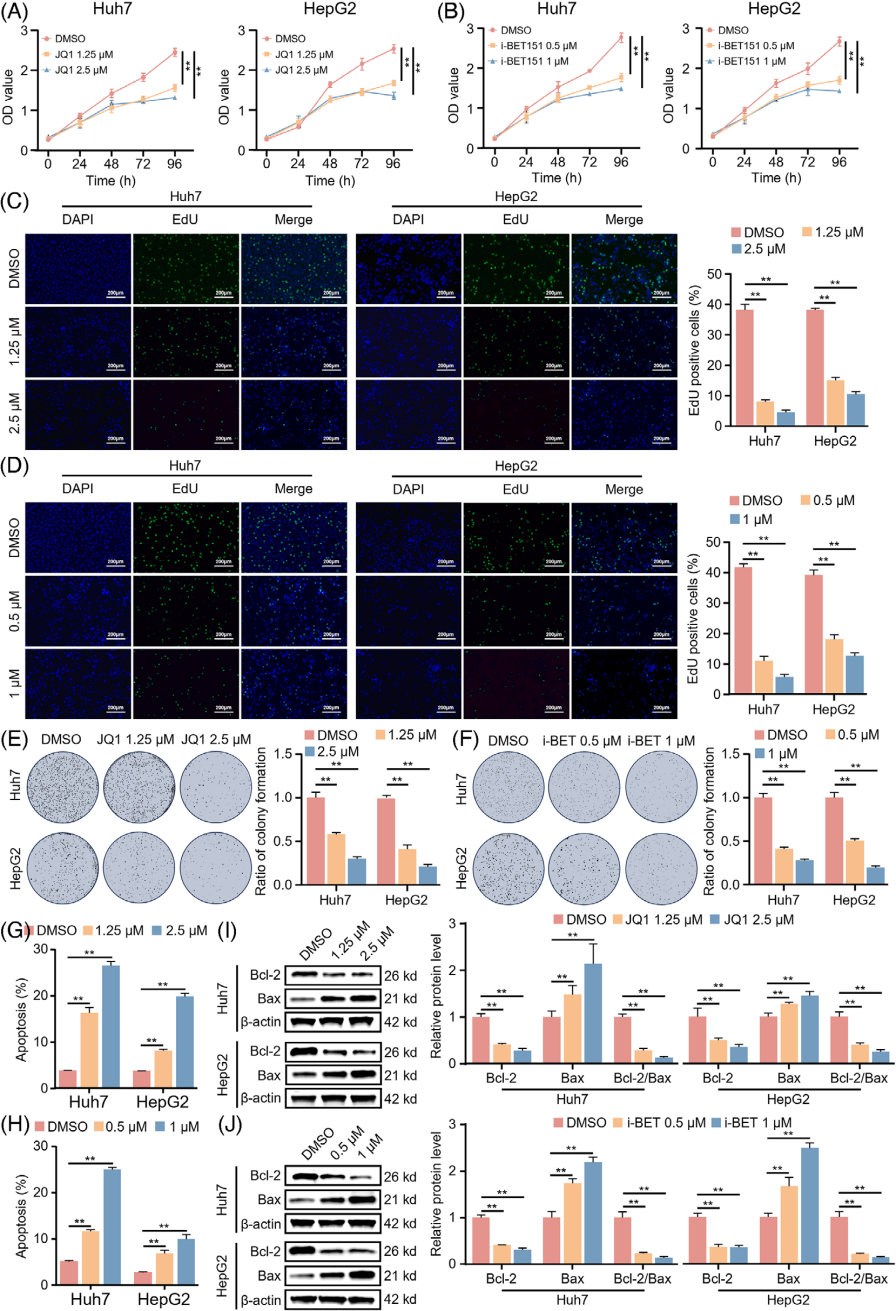
Targeting super‐enhancers (SEs) inhibits hepatocellular carcinoma (HCC) cell proliferation. (A and B) The viability of Huh7 and HepG2 cells treated with JQ‐1 (1.25 and 2.5 µM) or i‐BET151 (.5 and 1.0 µM) was measured at different time points using the CCK‐8 assay. (C and D) The proliferative capacity of Huh7 and HepG2 cells treated with JQ‐1 (1.25 and 2.5 µM) or i‐BET151 (.5 and 1.0 µM) for 48 h was assessed using the 5‑ethynyl‑2′‑deoxyuridine (EdU) assay. Scale bar: 200 µm. (E and F) The ability of JQ‐1 (1.25 and 2.5 µM) or i‐BET151 (.5 and 1.0 µM) to inhibit the proliferation of Huh7 and HepG2 cells were evaluated by colony formation assay. (G and H) The apoptosis rate of Huh7 and HepG2 cells treated with JQ‐1 (1.25 and 2.5 µM) or i‐BET151 (.5 and 1.0 µM) for 48 h was determined by flow cytometry. (I and J) The protein levels of Bcl‐2 and Bax in Huh7 and HepG2 cells treated with JQ‐1 (1.25 and 2.5 µM) or i‐BET151 (.5 and 1.0 µM) for 48 h were analysed by Western blotting. Data represent the mean ± SD; ^**^ *p* < .01.

This study first assessed the phenotypic consequences of targeting SEs in HCC cells using SE inhibitors. CCK‐8 assays revealed that JQ‐1 (Figure 1A) and i‐BET151 (Figure 1B) significantly suppressed the viability of Huh7 and HepG2 cells. Furthermore, EdU assay results indicated that treatment with JQ‐1 (Figure 1C) and i‐BET151 (Figure 1D) markedly diminished fluorescence intensity in Huh7 and HepG2 cells relative to controls, demonstrating a marked inhibition of cell proliferation. Similarly, the colony‐forming ability of Huh7 and HepG2 cells treated with JQ‐1 (Figure 1E) and i‐BET151 (Figure 1F) was markedly reduced compared with that of controls. The impact of SE inhibitors on the apoptotic capacity of HCC cells was further assessed. Flow cytometry results revealed that treatment with JQ‐1 (Figures 1G and S1A) and i‐BET151 (Figures 1H and S1B) significantly increased the rate of apoptosis. Consistently, Western blotting (WB) analysis demonstrated that treatment with JQ‐1 (Figure 1I) and i‐BET151 (Figure 1J) decreased the expression of B‐cell lymphoma 2 (Bcl‐2) and elevated that of Bcl‐2 associated X protein (Bax), thereby significantly reducing the Bcl‐2/Bax ratio. The observation that targeting SEs suppressed proliferation and induced cell death highlights their crucial role in HCC.

### 3.2 KIAA1522 is a key SE‐driven gene in HCC

**FIGURE 2:**
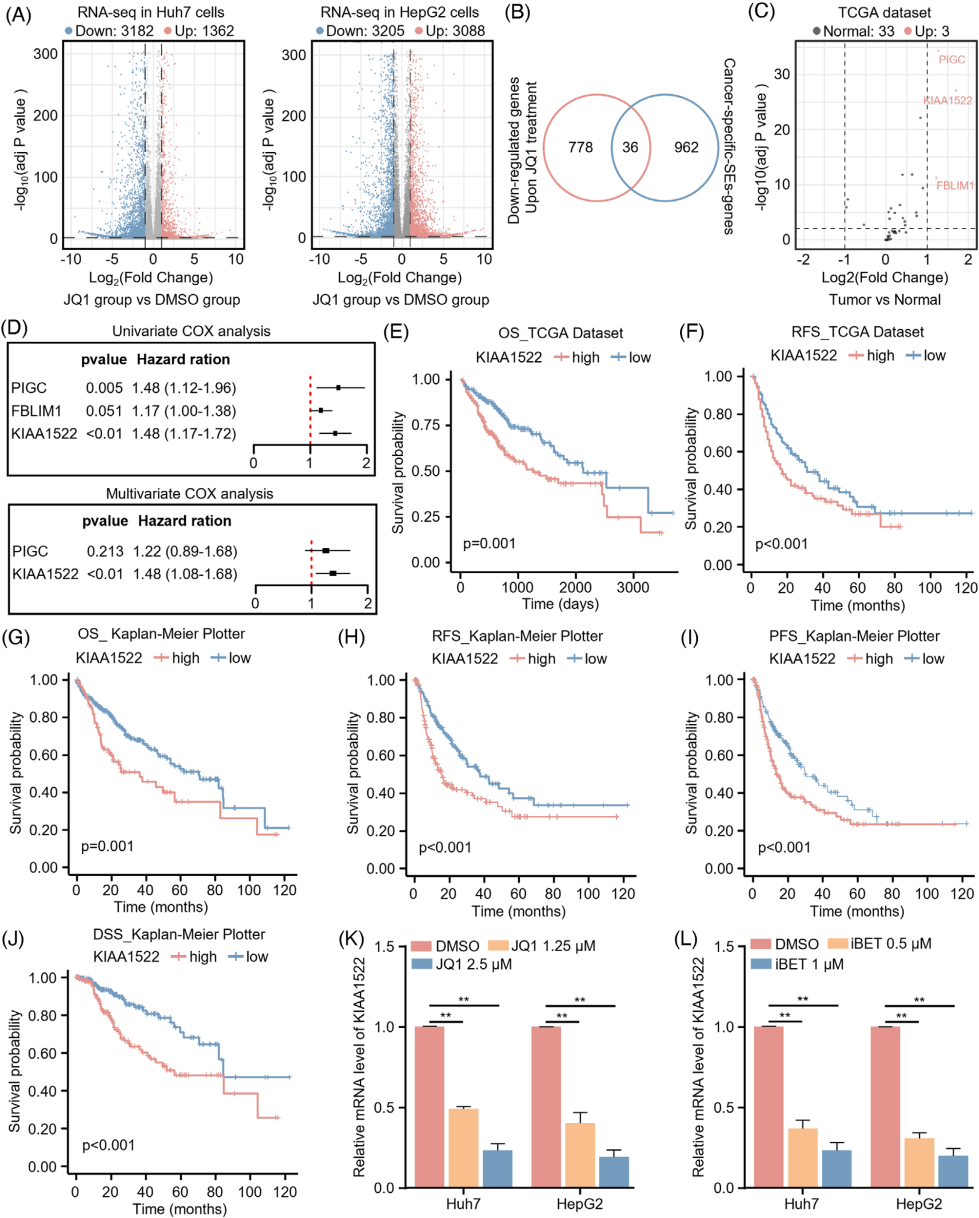
Identification of KIAA1522 as a hepatocellular carcinoma (HCC)‑specific super‐enhancer (SE) gene. (A) Volcano plot displaying the differential expression analysis of RNA sequencing (RNA‐seq) in Huh7 and HepG2 cell treated with 2.5 µM JQ‐1 for 48 h. (B) Intersection analysis of downregulated differentially expressed genes (DEGs) and HCC‐specific SE genes. (C) Volcano plot displaying the differential expression analysis of HCC‐specific SE genes based on The Cancer Genome Atlas (TCGA) dataset. (D) Forest plot of univariate and multivariate COX regression analyses for HCC‐specific SE genes in TCGA cohort. (E and F) Kaplan‒Meier curves of overall survival (OS) and recurrence‐free survival (RFS) for patients with high versus low KIAA1522 expression in TCGA dataset. (G‒J) Kaplan‒Meier curves of OS, RFS, progression‐free survival (PFS) and disease‐specific survival (DSS) for patients with high versus low KIAA1522 expression in the Kaplan‒Meier Plotter database. (K and L) Relative KIAA1522 mRNA levels in Huh7 and HepG2 cells treated with JQ‐1 (1.25 and 2.5 µM) or i‐BET151 (.5 and 1.0 µM) for 48 h were determined by quantitative reverse transcription PCR (qRT‐PCR). Data represent the mean ± SD; ^**^ *p* < .01.

To further explore the transcriptional regulation of HCC genes by SEs, RNA‐seq was carried out on Huh7 and HepG2 cells exposed to JQ‐1. Differential analysis identified 4544 and 6293 DEGs in these cell lines, respectively (Figure 2A). Based on HCC‐specific SE‐associated genes obtained from the SEdb 3.0 database, intersection with consistently downregulated DEGs revealed 36 HCC‐specific SE‐associated genes that showed significantly low expression after treatment with JQ‐1 (Figure 2B). Furthermore, in TCGA HCC cohort, phosphatidylinositol glycan anchor biosynthesis class C (PIGC), filamin‐binding LIM protein 1 (FBLIM1) and KIAA1522 were significantly overexpressed in cancerous versus normal tissues (Figure 2C). Subsequently, the prognostic significance of these three candidates in HCC was analysed. Univariate COX regression ascertained that PIGC and KIAA1522 expression was associated with HCC clinical outcomes (Figure 2D). Multivariate COX regression (Figure 2D) confirmed that only KIAA1522 represented an independent prognostic marker. Importantly, survival analysis showed that in TCGA database (Figure 2E,F), high expression of KIAA1522 was associated with poorer OS and RFS; in the Kaplan‒Meier Plotter database (Figure 2G‒J), high KIAA1522 expression correlated with worse OS, RFS, PFS and DSS. Finally, qRT‐PCR validation confirmed that treatment with JQ‐1 (Figure 2K) and i‐BET151 (Figure 2L) greatly diminished KIAA1522 mRNA levels. Considering these results, KIAA1522 was selected as the focus for subsequent in‐depth investigation in this study.

### 3.3 KIAA1522 is upregulated in HCC tissues

**FIGURE 3:**
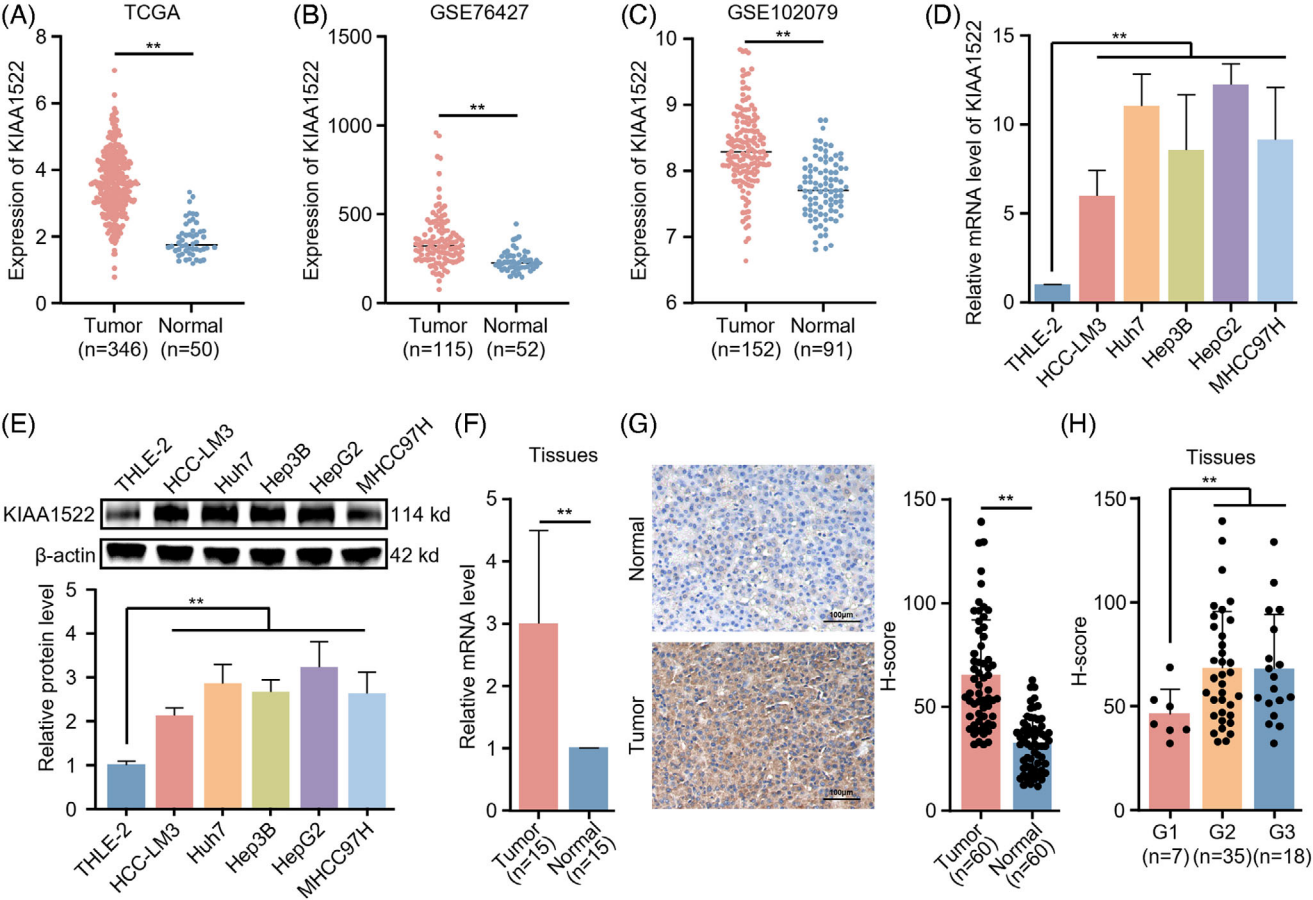
KIAA1522 is upregulated in hepatocellular carcinoma (HCC) tissues. (A‒C) Expression analysis of KIAA1522 in HCC and normal tissues using The Cancer Genome Atlas (TCGA), GSE76427 and GSE102079 datasets. (D and E) The mRNA and protein levels of KIAA1522 in hepatocytes and various HCC cell lines were detected by quantitative reverse transcription PCR (qRT‐PCR) and Western blotting, respectively. (F) Relative mRNA expression of KIAA1522 between tumour and paired normal tissues in HCC patients (*n* = 15) was assessed via qRT‑PCR. (G) Representative immunohistochemistry (IHC) images of KIAA1522 expression in real‐world HCC tumours and paired normal tissues (*n* = 60). Scale bar: 100 µm. (H) Statistical analysis of KIAA1522 *H*‐scores across different tumour differentiation grades. Data are mean ± SD; ^**^ *p* < .01.

Analysis of three independent HCC datasets (TCGA, GSE76427 and GSE102079) disclosed that KIAA1522 was markedly upregulated in HCC versus non‐malignant tissues (Figure 3A‒C). Additionally, KIAA1522 mRNA and protein were pronouncedly higher in HCC cell lines versus THLE‐2 (Figure 3D,E). In parallel, analysis of clinical specimens revealed markedly heightened KIAA1522 mRNA (qRT‑PCR, *n* = 15, Figure 3F) and protein (IHC, *n* = 60, Figure 3G) levels in HCC versus normal liver tissues. Moreover, subgroup analysis demonstrated that KIAA1522 protein exhibited pronounced upregulation in poorly differentiated (G2‒G3) versus well‐differentiated (G1) HCC tissues (Figure 3H). Collectively, the findings indicated that KIAA1522 is upregulated and linked to poor differentiation in HCC.

### 3.4 Identification of the core SE region driving KIAA1522 transcription

**FIGURE 4:**
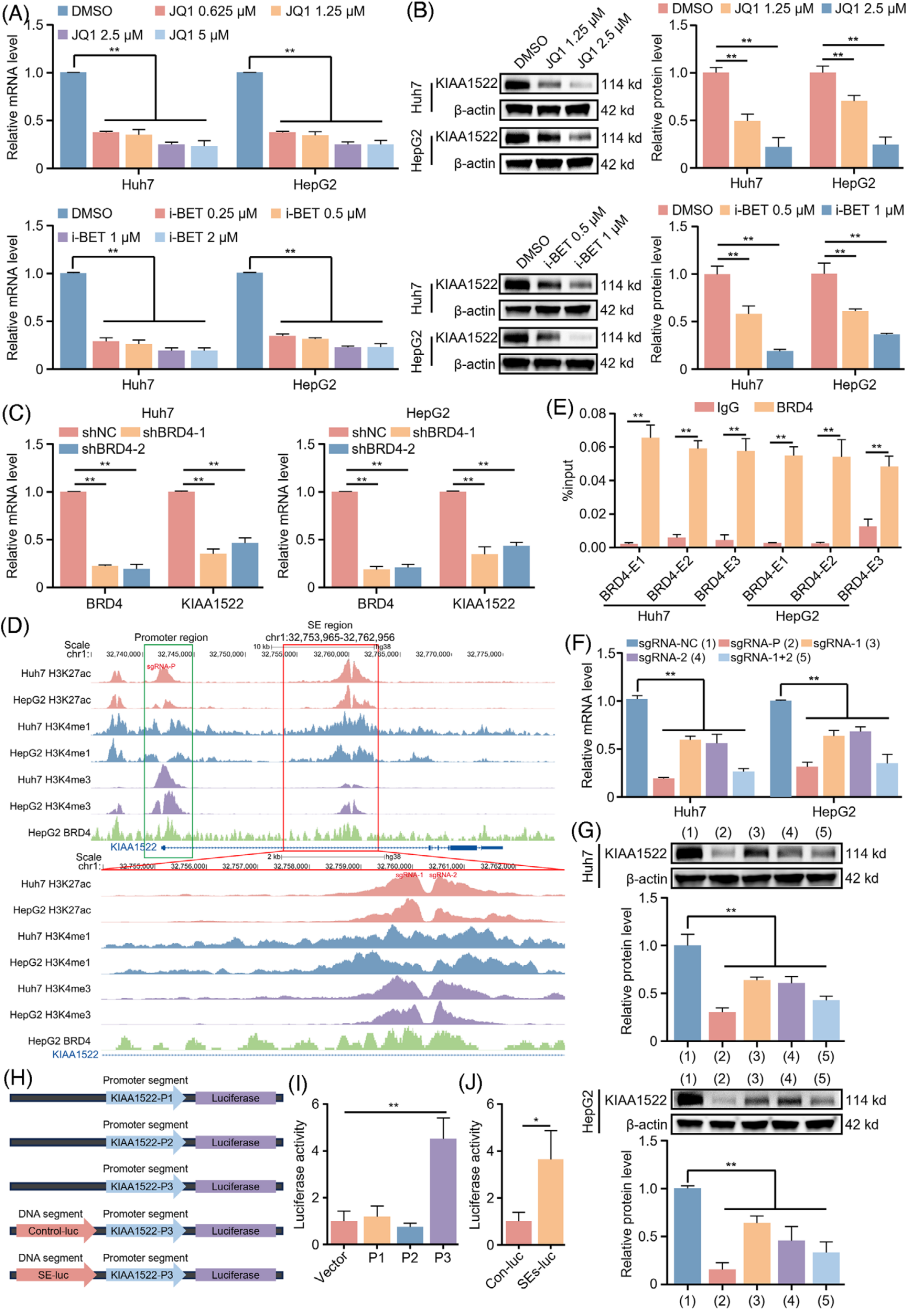
Core super‐enhancer (SE) region facilitates the transcriptional activation of KIAA1522. (A and B) The mRNA and protein levels of KIAA1522 in Huh7 and HepG2 cells treated with JQ‐1 or i‐BET151 for 48 h were detected by quantitative reverse transcription PCR (qRT‐PCR) and Western blotting, respectively. (C) The mRNA levels of bromodomain‐containing protein 4 (BRD4) and KIAA1522 in Huh7 and HepG2 after BRD4 knockdown were detected by qRT‐PCR. (D) Tracks showing the H3K27ac, H3K4me1 and H3K4me3 ChIP‐seq at the KIAA1522 locus in Huh7 and HepG2 cells, with BRD4 additionally shown in HepG2 cells. The regions for the KIAA1522 promoter, putative core SEs and sgRNAs of CRISPRi are presented. (E) ChIP‐qPCR analysis of BRD4 enrichment in the SEs and promoter regions of KIAA1522. (F and G) The mRNA and protein levels of KIAA1522 in Huh7 and HepG2 cells were analysed by qRT‐PCR and Western blotting, respectively, following suppression of SEs by the CRISPRi system with targeted sgRNAs. (H) Schematic diagram of the KIAA1522 promoter and SE luciferase reporter constructs. (I and J) The regulatory connection between the core SE region and the strongest transcriptionally active promoter region of KIAA1522 (KIAA1522‐P3) was investigated using a dual‐luciferase reporter assay. Data represent the mean ± SD; ^*^ *p* < .05, ^**^ *p* < .01.

To assess the SE‐driven transcriptional activity of KIAA1522, Huh7 and HepG2 cells were cultured with SE inhibitors. This treatment diminished KIAA1522 mRNA (Figure 4A) and protein (Figure 4B) expression. Subsequently, shRNAs targeting BRD4 were employed to investigate whether BRD4 participates in the transcriptional regulation of KIAA1522. As shown in Figure 4C, knockdown of BRD4 downregulated the mRNA level of KIAA1522, indicating that KIAA1522 exhibits typical features of a gene regulated by SEs.

To precisely identify the SE regions that regulate the expression of KIAA1522 in HCC, we analysed publicly available H3K27ac ChIP‐seq profiles from Huh7 and HepG2 cells (Figure 4D). This analysis revealed a continuous enrichment of H3K27ac signals within the predicted KIAA1522‐associated SE region (chr1: 32753965‒32762956). Furthermore, by examining H3K4me1 and H3K4me3 ChIP‐seq profiles in these cell lines, we delineated the promoter region. Specifically, enrichment of H3K27ac and H3K4me3 within chr1: 32741365‒32742714 led us to designate it as the KIAA1522 promoter region. To characterise the SEs and promoter loci of KIAA1522, we inspected publicly available BRD4 ChIP‐seq profiles from HepG2 cells. This analysis revealed co‑enrichment of BRD4 at both the KIAA1522 promoter and the predicted common SE region. Notably, ChIP‑qPCR validation in Huh7 and HepG2 cells confirmed BRD4 occupancy at multiple sites within these regions (Figure 4E).

To validate the direct transcriptional regulatory role of SE regions on KIAA1522, a CRISPR interference (CRISPRi) system was specifically engineered, comprising two SE‐targeting sgRNAs (sgRNA‐1 and ‐2) and a promoter‐targeting positive control (sgRNA‐P). This system was used to recruit dCas9‐KRAB, which disrupts interactions between SEs and the promoter. Results showed that targeting either the SEs or the promoter region of KIAA1522 significantly suppressed KIAA1522 mRNA and protein expression (Figure 4F,G), demonstrating that SEs regulate KIAA1522 expression. Furthermore, a luciferase reporter assay determined whether SEs enhance KIAA1522 promoter activity. Three 450‐bp segments (KIAA1522‐P1, ‐P2 and ‐P3) were inserted into the pGL3.0‐basic, and the segment demonstrating the greatest effectiveness was selected for SE activity testing (Figure 4H,I). Subsequently, a predicted active SE segment and a negative control segment were inserted upstream of the KIAA1522‐P3 promoter region. Compared with the control‐luc group, the SEs‐luc group exhibited significantly enhanced luciferase activity (Figure 4J). These results demonstrate that the SE region located at chr1: 32753965‒32762956 potentially interacts with the KIAA1522 promoter to enhance its transcriptional activation.

### 3.5 Targeting KIAA1522 impedes HCC progression

**FIGURE 5:**
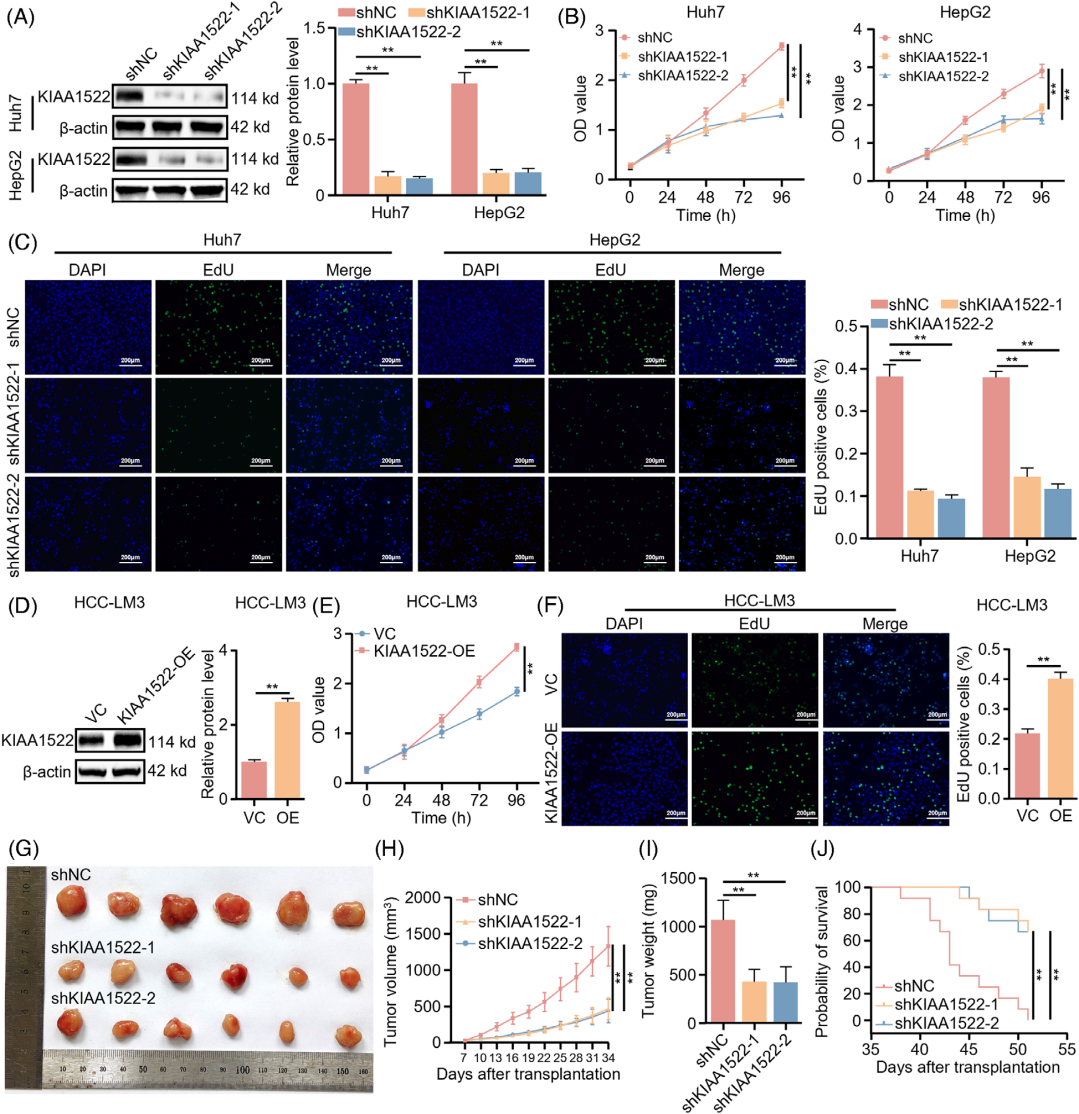
KIAA1522 knockdown suppresses hepatocellular carcinoma (HCC) cell proliferation. (A) Changes in KIAA1522 protein expression levels in Huh7 and HepG2 cells following KIAA1522 knockdown were detected by Western blotting. (B and C) The viability and proliferative capacity of Huh7 and HepG2 cells following KIAA1522 knockdown were measured by CCK‐8 and 5‑ethynyl‑2′‑deoxyuridine (EdU) assays. Scale bar: 200 µm. (D) Changes in KIAA1522 protein expression levels in HCC‐LM3 cells following KIAA1522 overexpression were detected by Western blotting. (E and F) The viability and proliferative capacity of HCC‐LM3 cells following KIAA1522 overexpression were measured by CCK‐8 and EdU assays. Scale bar: 200 µm. (G) The xenografts were isolated from nude mice inoculated with HepG2 cells stably expressing shNC, shKIAA1522‐1 and shKIAA1522‐2 (*n* = 6/group). (H) Changes in tumour volume during xenograft development (*n* = 6/group). (I) Statistical analysis of xenograft weight (*n* = 6/group). (J) Survival curve showing the survival status of tumour‐bearing mice in the shNC, shKIAA1522‐1 and shKIAA1522‐2 groups (*n* = 12/group). Data represent the mean ± SD; ^**^ *p* < .01.

For functional investigation of KIAA1522 in HCC, KIAA1522‐knockdown Huh7 and HepG2 cells were generated (Figures 5A and S2A). As expected, KIAA1522 knockdown pronouncedly impaired Huh7 and HepG2 cell viability, proliferation, migration and invasion (Figures 5B,C and S2B,C). In parallel, KIAA1522‐overexpressing HCC‐LM3 cells were generated for the functional rescue experiment (Figures 5D and S2D). Functional assays demonstrated that exogenous KIAA1522 overexpression significantly promoted the viability (Figure 5E), proliferation (Figure 5F), migration (Figure S2E) and invasion (Figure S2F) of HCC‐LM3 cells. Collectively, these observations suggest that KIAA1522 is indispensable for promoting malignant biological processes in vitro.

Furthermore, xenograft tumours were developed using nude mice bearing KIAA1522‐knockdown HepG2 cells to elucidate the function of KIAA1522 in vivo. KIAA1522 knockdown significantly inhibited the growth of xenograft tumours (Figure 5G‒I) and prolonged survival time (Figure 5J) compared with controls, indicating that targeting KIAA1522 in vivo can suppress HCC tumour progression.

### 3.6 Inhibition of KIAA1522 induces ferroptosis in HCC

**FIGURE 6:**
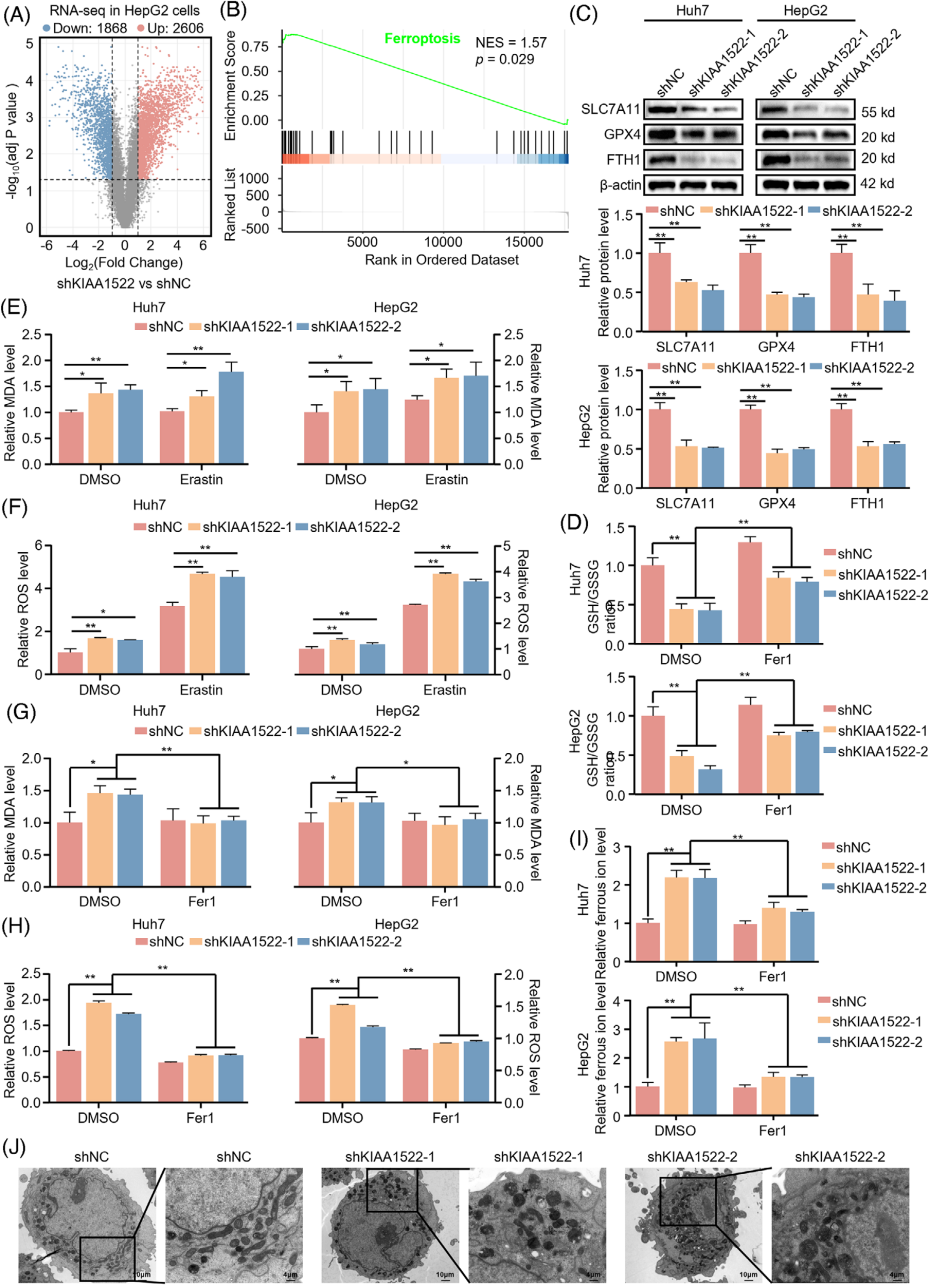
Inhibition of KIAA1522 induces ferroptosis in hepatocellular carcinoma (HCC). (A) Volcano plot illustrating differentially expressed genes (DEGs) of RNA sequencing (RNA‐seq) data in HepG2 cells following shNC or shKIAA1522 intervention. (B) Gene Set Enrichment Analysis (GSEA) revealed a significant enrichment of the ferroptosis pathway. (C) The protein levels of solute carrier family 7 member 11 (SLC7A11), glutathione peroxidase 4 (GPX4) and ferritin heavy chain 1 (FTH1) in Huh7 and HepG2 cells following KIAA1522 knockdown were detected by Western blotting. (D) The GSH/GSSG ratio in KIAA1522‐knockdown Huh7 and HepG2 cells with or without Ferrostatin‐1 (Fer‐1) (2 µM) treatment for 24 h was determined using the GSH/GSSG assay kit. (E) The malondialdehyde (MDA) levels in KIAA1522‐knockdown Huh7 and HepG2 cells with or without Erastin (10 µM) treatment for 24 h was determined using the MDA assay kit. (F) The reactive oxygen species (ROS) levels in KIAA1522‐knockdown Huh7 and HepG2 cells with or without Erastin (10 µM) treatment for 24 h were determined by flow cytometry. (G) The MDA levels in KIAA1522‐knockdown Huh7 and HepG2 cells with or without Fer‐1 (2 µM) treatment for 24 h was determined using the MDA assay kit. (H) The ROS levels in KIAA1522‐knockdown Huh7 and HepG2 cells with or without Fer‐1 (2 µM) treatment for 24 h were determined by flow cytometry. (I) The ferrous iron levels in KIAA1522‐knockdown Huh7 and HepG2 cells with or without Fer‐1 (2 µM) treatment for 24 h was determined using the ferrous ion assay kit. (J) Representative electron micrographs in HepG2 cells following shNC or KIAA1522‐knockdown (shKIAA1522‐1 and shKIAA1522‐2) intervention were captured using transmission electron microscopy. Narrowed view, scale bar: 10 µm; enlarged view, scale bar: 4 µm. Data represent the mean ± SD; ^**^ *p* < .01.

Next, we investigated the pro‐tumour mechanism of KIAA1522 in HCC. We performed RNA‐seq on KIAA1522‐knockdown HepG2 cells, which revealed 1868 downregulated and 2606 upregulated DEGs compared with control group (Figure 6A). GSEA indicated significant activation of the ferroptosis pathway (Figure 6B). Subsequently, we examined the expression of ferroptosis‐related proteins after KIAA1522 intervention. Loss‐of‐function experiments demonstrated that KIAA1522 knockdown markedly reduced the levels of SLC7A11, glutathione peroxidase 4 (GPX4) and ferritin heavy chain 1 (FTH1) (Figure 6C). Conversely, KIAA1522 overexpression notably increased the expression levels of these proteins (Figure S3A).

Relevant markers were further assessed to validate the association between KIAA1522 and ferroptosis. Specifically, KIAA1522 knockdown lowered the GSH/GSSG ratio in HCC cells (Figure 6D), and this effect was reversed by treatment with Fer‐1 (Figure 6D) or KIAA1522 overexpression (Figure S3B). Furthermore, as expected, KIAA1522 knockdown significantly increased the levels of MDA and ROS in HCC cells and enhanced the effect of the ferroptosis inducer Erastin (Figure 6E,F); these effects were attenuated by treatment with Fer‐1 (Figure 6G,H) or KIAA1522 overexpression (Figure S3C,D). We also detected changes in intracellular ferrous ion levels. Specifically, KIAA1522 knockdown elevated them in HCC cells (Figure 6I), and this effect was reversed by treatment with Fer‐1 (Figure 6I) or KIAA1522 overexpression (Figure S3E). Importantly, transmission electron microscopy revealed that KIAA1522 knockdown induced mitochondrial shrinkage, membrane densification and cristae loss in HepG2 cells (Figure 6J) and Huh7 cells (Figure S3F). These results indicate that KIAA1522 promotes cell proliferation by inhibiting ferroptosis.

### 3.7 Inhibiting SE activity mimics KIAA1522 knockdown

**FIGURE 7:**
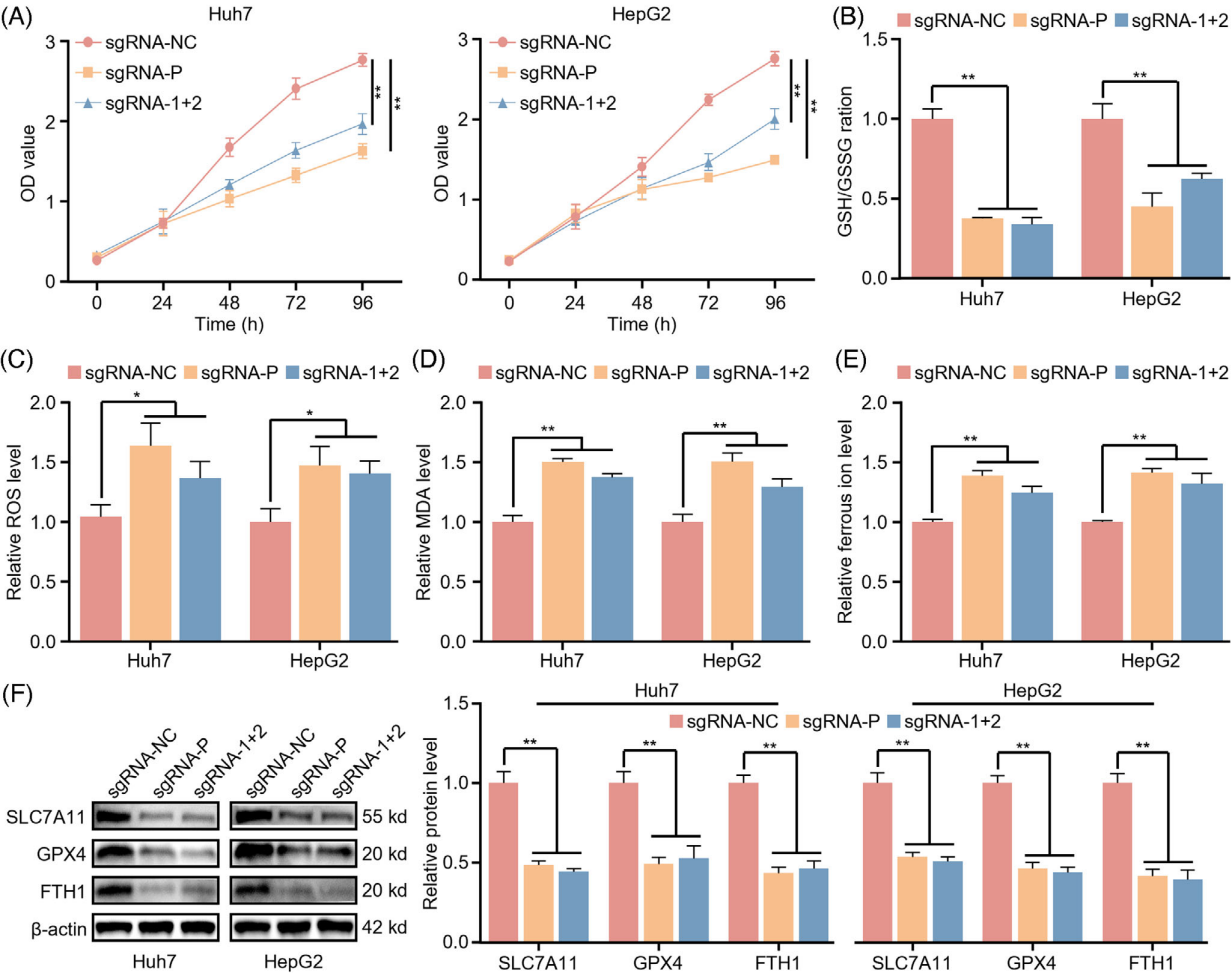
Inhibiting super‐enhancer (SE) activity mimics KIAA1522 knockdown. (A) The viability of Huh7 and HepG2 cells following inhibition of SEs through CRISPRi was assessed using the CCK‐8 assay. (B) The GSH/GSSG ratio in Huh7 and HepG2 cells following inhibition of SEs through CRISPRi was determined using the GSH/GSSG assay kit. (C) The reactive oxygen species (ROS) levels in Huh7 and HepG2 cells following inhibition of SEs through CRISPRi were determined by flow cytometry. (D) The malondialdehyde (MDA) levels in Huh7 and HepG2 cells following inhibition of SEs through CRISPRi were determined using the MDA assay kit. (E) The ferrous iron levels in Huh7 and HepG2 cells following inhibition of SEs through CRISPRi were determined using the ferrous ion assay kit. (F) The protein levels of solute carrier family 7 member 11 (SLC7A11), glutathione peroxidase 4 (GPX4) and ferritin heavy chain 1 (FTH1) in Huh7 and HepG2 cells following inhibition of SEs through CRISPRi were detected by Western blotting. Data represent the mean ± SD; ^*^ *p* < .05, ^**^ *p* < .01.

Given that the transcription of KIAA1522 is regulated by SEs, we investigated whether targeting SEs could mimic the biological functional changes induced by KIAA1522 knockdown. The sgRNA1+2+3 and sgRNA‐P were transfected into Huh7 and HepG2 cells. Targeting SEs significantly inhibited cell viability in CCK‐8 assays (Figure 7A). Importantly, SE targeting markedly suppressed the GSH/GSSG ratio (Figure 7B), with concomitant elevations observed in ROS, MDA and ferrous ion levels (Figure 7C‒E). Furthermore, SLC7A11, GPX4 and FTH1 were significantly downregulated in the SE‐targeting group (Figure 7F). Collectively, these data demonstrate that SEs crucially mediate the inhibition of ferroptosis by KIAA1522 to drive cell proliferation.

### 3.8 KIAA1522 knockdown activates ferroptosis through ATF4

**FIGURE 8:**
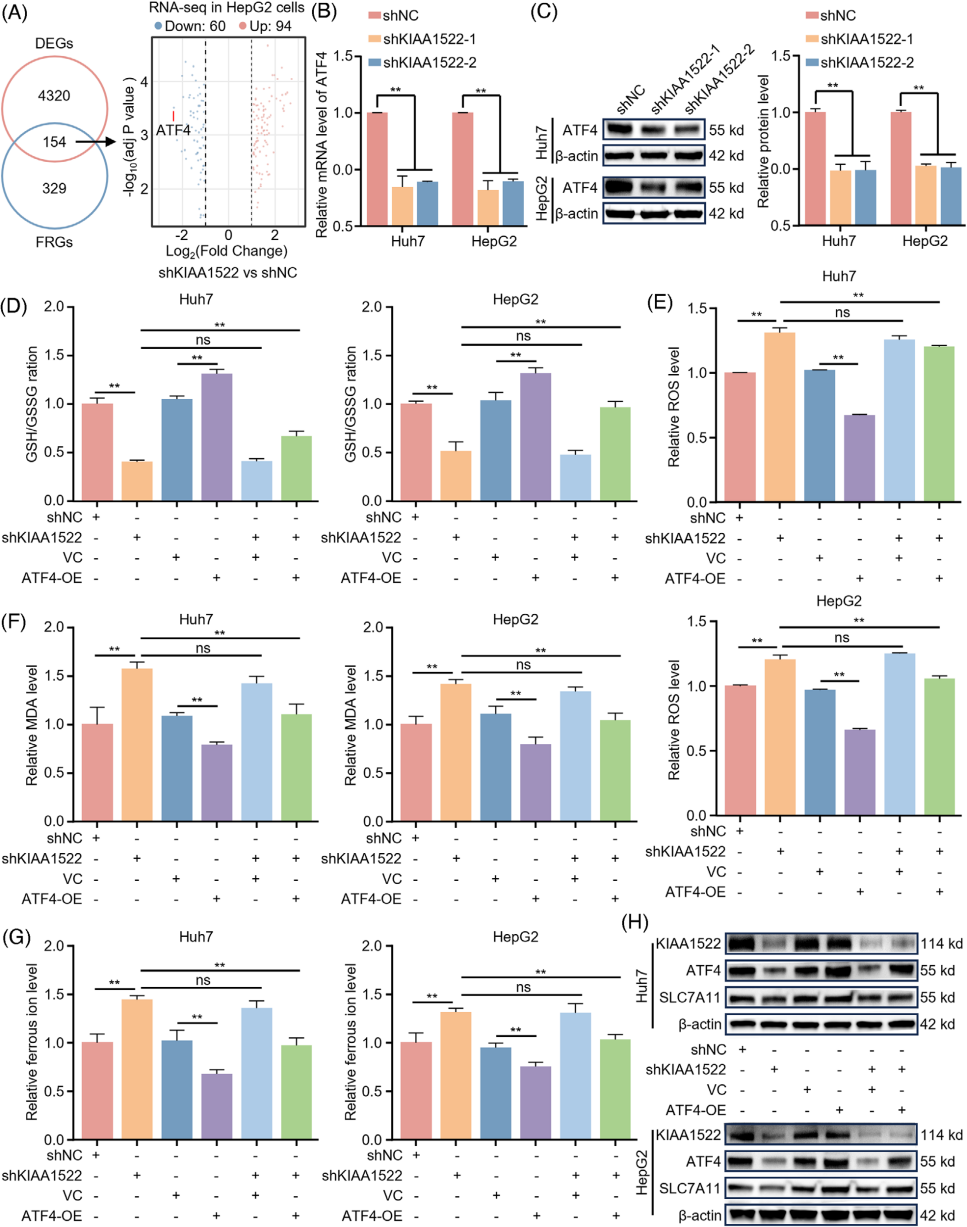
KIAA1522 knockdown activates ferroptosis through activating transcription factor 4 (ATF4). (A) Venn diagram and volcano plot illustrating that ATF4 was the most significantly downregulated ferroptosis‐related differentially expressed gene (DEG) in the RNA sequencing (RNA‐seq) data from HepG2 cells following KIAA1522 knockdown. (B and C) The mRNA and protein levels of ATF4 in Huh7 and HepG2 cells following KIAA1522 knockdown were analysed by quantitative reverse transcription PCR (qRT‐PCR) and Western blotting, respectively. (D) The GSH/GSSG ratio in Huh7 and HepG2 cells following KIAA1522 knockdown and/or ATF4 overexpression was determined using the GSH/GSSG assay kit. (E) The reactive oxygen species (ROS) levels in Huh7 and HepG2 cells following KIAA1522 knockdown and/or ATF4 overexpression were determined by flow cytometry. (F) The malondialdehyde (MDA) levels in Huh7 and HepG2 cells following KIAA1522 knockdown and/or ATF4 overexpression were determined using the MDA assay kit. (G) The ferrous iron levels in Huh7 and HepG2 cells following KIAA1522 knockdown and/or ATF4 overexpression were determined using the ferrous ion assay kit. (H) The protein levels of KIAA1522, solute carrier family 7 member 11 (SLC7A11) and ATF4 in Huh7 and HepG2 cells following KIAA1522 knockdown and/or ATF4 overexpression were detected by Western blotting. Data represent the mean ± SD; ^**^ *p* < .01, ns, not significant.

To further explore KIAA1522‐mediated ferroptosis, 483 ferroptosis‑related genes (FRGs) were retrieved from FerrDb.^23^ Next, intersection analysis was conducted between these FRGs and the DEGs identified from RNA‐seq data of KIAA1522‐knockdown cells. The results (Figure 8A) showed a set of overlapping genes between FRGs and DEGs, and the volcano plot of these intersecting genes indicated that ATF4 (a key regulator of ferroptosis) was significantly downregulated. Validation experiments confirmed that KIAA1522 knockdown suppressed ATF4 mRNA (Figure 8B) and protein (Figure 8C) levels, whereas KIAA1522 overexpression substantially increased ATF4 mRNA (Figure S4A) and protein (Figure S4B) levels.

Next, to verify whether ATF4 mediates the activation of ferroptosis induced by KIAA1522 knockdown, a rescue experiment was performed by overexpressing ATF4. Results from qRT‑PCR (Figure S4C) and WB (Figure S4D) confirmed that overexpression of ATF4 significantly elevated its mRNA and protein levels in Huh7 and HepG2 cells. Changes in ferroptosis‐related phenotypes are shown in Figure 8D‒G, where HCC cells with KIAA1522 knockdown alone exhibited a marked reduction in the GSH/GSSG ratio and a pronounced rise in ROS, MDA and ferrous ion levels, consistent with previous observations. In contrast, HCC cells overexpressing ATF4 alone showed a pronounced increase in the GSH/GSSG ratio, along with markedly decreased ROS, MDA and ferrous ion levels, suggesting that ATF4 acts as a critical regulator of ferroptosis. Importantly, ATF4 overexpression substantially reversed the ferroptosis‑related phenotypic changes caused by KIAA1522 knockdown. As reported in the literature, ATF4 is a transcription factor of SLC7A11. Consistently, dual‐luciferase assay showed that ATF4 overexpression enhanced SLC7A11 promoter activity, which was abolished by mutating the ATF4‐binding site (Figure S4E). Additionally, the expression of KIAA1522, ATF4 and SLC7A11 was assessed in the above cell groups. Consistently, KIAA1522 downregulation suppressed ATF4 and SLC7A11 expression, whereas ATF4 overexpression reversed this effect (Figures 8H and S4F). These findings indicate that ATF4 is required for the activation of ferroptosis in HCC following KIAA1522 knockdown.

## 4 DISCUSSION

The occurrence and progression of HCC involve complex genetic and epigenetic alterations.^24^, ^25^ Recent research has shown that SEs, as key epigenetic regulatory elements, are aberrantly activated in transcriptional reprogramming and oncogene dysregulation across various types of cancers.^26^, ^27^ Moreover, unlike their role in normal cells, SEs in tumour cells promote cell proliferation and are involved in distant metastasis, therapy resistance and immune evasion, among other malignant biological behaviours.^28^ A systematic review has implicated aberrant SE activation in HCC malignant progression, with mounting evidence demonstrating that SE redistribution promotes malignant transformation.^19^, ^29^ Consistent with these findings, the present study showed that SE inhibitors effectively suppressed HCC cell proliferation. Through multi‐omics analysis, we also identified the SE‐driven gene KIAA1522 as a novel oncogene abundantly expressed in HCC and linked to unfavourable prognosis. Furthermore, in‐depth exploration revealed that SE‐induced transcriptional activation of KIAA1522 grants HCC cells resistance to ferroptosis via the ATF4/SLC7A11 pathway, thereby promoting HCC progression. This finding illuminates the specificity of epigenetic governance in HCC and provides a novel mechanistic perspective on SE‐mediated malignant phenotypes.

Research indicates that inhibiting SEs can effectively suppress tumour cell malignancy and induce cell death.^29^, ^30^ This study validated that SE inhibitors curb HCC cell growth, suggesting the presence of dysregulated SEs in HCC cells that are targeted by these inhibitors. To identify potential SE‐regulated oncogenes in HCC, RNA‐seq was performed on HCC cells treated with JQ‐1. Through differential expression analysis combined with evaluation of HCC‐specific SE genes, three candidate genes (PIGC, FBLIM1 and KIAA1522) were identified. Subsequent univariate and multivariate COX regression analysis and survival prognosis assessment based on TCGA‐HCC dataset revealed KIAA1522 as an independent prognostic risk factor significantly associated with poorer survival outcomes in HCC, establishing it as the key gene for this study. Upregulation of KIAA1522 is consistently reported in multiple malignancies and parallels unfavourable clinical outcomes; moreover, it is involved in key pathways including mechanistic target of rapamycin (mTOR), extracellular signal‐regulated kinases (ERK), Wnt and tumour necrosis factor‐alpha (TNFα), thereby promoting oncogenic proliferation, resistance to therapy, epithelial‒mesenchymal transition and metastasis.^31^, ^32^, ^33^, ^34^ Although research on KIAA1522 in HCC is limited, existing evidence suggests that its abnormal expression is linked to disease progression. Studies have detected elevated KIAA1522 expression in HCC,^35^ implicating its involvement in Wnt/catenin beta‐1 (β‐catenin) activation.^34^, ^36^ The present study assessed the role of KIAA1522 by integrating three independent HCC datasets (TCGA, GSE76427 and GSE102079), HCC cell lines and real‐world HCC tissues. Consistent with prior research findings, this investigation confirmed high expression of KIAA1522 in HCC, with higher levels observed in poorly or undifferentiated tumour cells. Importantly, in vitro functional experiments revealed that reducing KIAA1522 expression inhibits HCC cell proliferation, migration and invasion, while overexpression promotes malignant phenotypes. More significantly, in a nude mouse xenograft model, KIAA1522 knockdown markedly suppressed tumour growth and prolonged survival time. Therefore, as a critical oncogenic factor, KIAA1522 contributes to HCC malignancy, representing a novel and promising target for intervention with potential for clinical translation.

Furthermore, the upstream regulatory mechanisms underlying the abnormal overexpression of the oncogene KIAA1522 in HCC, especially epigenetic regulation, remain unclear. We found that SE inhibitors markedly curtailed KIAA1522 expression, and BRD4 knockdown yielded consistent results. Moreover, the SEs and promoter elements of KIAA1522 exhibited pronounced BRD4 enrichment in ChIP‐seq and ChIP‐qPCR profiles, which precisely mapped the SE region (chr1: 32753965‒32762956) responsible for regulating KIAA1522. Notably, targeting this SE region dramatically blunted KIAA1522 expression, confirming that SE dysregulation drives the sustained high expression of KIAA1522 in HCC. Through the present evidence, we epigenetically establish that SE activation acts as the upstream trigger for sustained KIAA1522 overexpression in HCC.

Ferroptosis executes intrinsic tumour‐suppressive programs, with canonical hallmarks including GSH exhaustion, ROS surplus, etc.^37^, ^38^, ^39^, ^40^ In this study, GSEA analysis of RNA‐seq data from KIAA1522‐knockdown cells revealed significant enrichment of the ferroptosis pathway. Subsequent in vitro experiments demonstrated GSH depletion, accumulation of ROS, MDA and ferrous ions, as well as typical mitochondrial morphological changes in KIAA1522‐knockdown HCC cells. Furthermore, KIAA1522 knockdown boosted the susceptibility of HCC cells to ferroptosis activators, whereas treatment with ferroptosis inhibitors or overexpression of KIAA1522 mitigated GSH depletion and overload of ROS, MDA and ferrous ions. These findings support the role of KIAA1522 as a dominant driver of ferroptosis. Tumour cells harbour complex regulatory networks, and emerging evidence indicates that KIAA1522 may influence sensitivity to ferroptosis through multiple signalling pathways. It has been reported that KIAA1522 participates in the mTOR pathway in colorectal cancer.^31^ Of note, mTOR can exert anti‐ferroptosis effects by promoting GPX4 protein synthesis and inhibiting detrimental autophagy.^41^ In HCC, KIAA1522 potentiates Wnt/β‐catenin activity,^34^, ^36^ which promotes SLC7A11 expression to ensure glutathione synthesis, thereby suppressing ferroptosis.^42^ Additionally, KIAA1522 potentiates TNFα‒nuclear factor‐κB (NFκB) signalling in lung adenocarcinoma.^32^ Notably, it has been demonstrated that TNFα reduces lipid peroxidation by upregulating FTH1 expression.^43^ Furthermore, KIAA1522 activates ERK signalling in esophageal cancer.^33^ Evidence has shown that ERK can potentiate the biological effects of multiple ferroptosis‐related pathways.^44^ Given that KIAA1522 transcription is regulated by SEs, this study found that using CRISPRi to target SEs successfully recapitulated the core ferroptosis phenotypes induced by KIAA1522 knockdown. This finding directly demonstrates that SE‐driven transcriptional activation of KIAA1522 represents an upstream epigenetic regulatory mechanism for resistance to ferroptosis in HCC cells. Notably, SEs have recently been implicated in the regulation of ferroptosis across multiple cancer types. In hepatoblastoma, it has been demonstrated that activation of SE‐driven tribbles pseudokinase 2 (TRIB2) expression suppresses ferroptosis via nuclear factor erythroid 2‐related factor 2 (NRF2) stabilisation.^45^ For lung adenocarcinoma, it has been reported that SE‐mediated upregulation of myeloma overexpressed (MYEOV) inhibits ferroptosis through GPX4 autophagic degradation.^46^ These findings, together with our identification of the SEs/KIAA1522 axis in HCC, suggest that SEs serve as a common epigenetic platform coordinating resistance to ferroptosis by driving distinct downstream targets in different tumour contexts.

Mechanistically, we discovered that the transcription factor ATF4 serves as a critical bridge linking KIAA1522 to ferroptosis. Specifically, it has been established by multiple studies that ATF4 directly binds and activates the promoter of the SLC7A11 gene; this effect promotes cystine uptake and GSH synthesis, thereby enhancing cellular resistance to ferroptosis.^47^, ^48^, ^49^ Analysis of RNA‐seq data from KIAA1522‐knockdown cells in this study identified ATF4 as the most significantly downregulated FRG. Cellular experiments confirmed decreased ATF4 expression in KIAA1522‐knockdown cells, while its levels increased in KIAA1522‐overexpressing cells. Functionally, overexpression of ATF4 significantly reversed the ferroptosis phenotypes induced by KIAA1522 knockdown, including restoration of the GSH/GSSG ratio and reduction in ROS, MDA and ferrous ion levels, as well as rescued SLC7A11 protein expression. Conversely, knockdown of KIAA1522 alone led to the concurrent downregulation of ATF4 and SLC7A11 expression. Therefore, ATF4 is a key effector molecule responsible for KIAA1522‑dependent resistance to ferroptosis. Notably, the transcriptional activation of SLC7A11 is not solely regulated by ATF4. Previous studies have demonstrated that NRF2 regulates SLC7A11 transactivation by interacting with the antioxidant response element (ARE) within its promoter.^50^ Consistently, the NRF2‐mediated antioxidant defense has been demonstrated to suppress ferroptosis in the context of bone cancer pain.^51^ In HCC, NRF2 exerts a pivotal function in resisting ferroptosis by activating SLC7A11, GPX4 and other target genes.^52^ Interestingly, ATF4 and NRF2 may exert synergistic modulation on SLC7A11 transcription by recognising the amino acid response element (AARE) and ARE within its promoter, respectively, and coordinately maintain high SLC7A11 expression under conditions of oxidative stress or amino acid deprivation.^49^ Although this study focused on ATF4, SLC7A11 plays a pivotal role in the regulation of ferroptosis and is co‐regulated by multiple transcription factors. Thus, we speculate that KIAA1522 may indirectly modulate NRF2 pathway activity through the regulation of ATF4 expression, or form a complementary defense network with the NRF2 pathway. Furthermore, whether KIAA1522 participates in the regulation of ferroptosis through other mechanisms represents a scientific question worthy of in‐depth investigation. Future studies should further explore the interplay between KIAA1522, ATF4 and NRF2, and elucidate the mechanism through which they coordinately regulate sensitivity to ferroptosis in HCC cells under different stress conditions.

This study has several limitations. First, the functional experiments primarily relied on HCC cell lines and nude mouse xenograft models, which cannot fully replicate the complexity of the human tumour microenvironment. Specifically, only subcutaneous xenografts derived from HepG2 cells were used, while orthotopic HCC models or patient‐derived xenograft (PDX) models, which better recapitulate the clinical features and tumour microenvironment of HCC, were not employed. Future studies should validate the in vivo function of KIAA1522 using these more physiologically relevant models to enhance clinical translation. Second, although ATF4 was identified as a key downstream mediator through which KIAA1522 inhibits ferroptosis, the potential synergy or compensation between ATF4 and NRF2 in regulating ferroptosis was not experimentally addressed, and the possibility of other parallel pathways remains open. Finally, the clinical translational value of KIAA1522 requires further validation through multicenter prospective cohort studies.

## 5 CONCLUSIONS

In summary, this study systematically identified and validated KIAA1522 as a key oncogene in HCC that is driven by SEs and holds independent prognostic value. It confers resistance to ferroptosis in HCC cells through the ATF4/SLC7A11 axis, thereby promoting tumour progression. These findings offer a novel perspective on the epigenetic regulatory network in HCC and lay a conceptual framework for clinical interventions directed at the SEs/KIAA1522/ferroptosis axis.

## AUTHOR CONTRIBUTIONS

Jiachun Sun contributed to the study conception and design, and supervised the study. Xinyang Li, Siyuan Hu, Haolin Shi, Pan Bian and Ziming Wang performed the experiments and analysed the data. Xinyang Li and Jiachun Sun wrote the manuscript. All the authors read and approved the final manuscript submitted.

## CONFLICT OF INTEREST STATEMENT

The authors declare they have no conflicts of interest.

## ETHICS STATEMENT

The study protocol was approved by the institutional ethics committee of the First Affiliated Hospital of Henan University of Science and Technology (K‐2025‐B013) in accordance with Declaration of Helsinki. Written informed consent was obtained from all participants. The animal experiments were approved by the Experimental Animal Ethics Committee of the First Affiliated Hospital of Henan University of Science and Technology (D‐2025‐B012).

## Supporting information



Supporting Information

## Data Availability

All data included in this study are available by the corresponding author upon request.
