# Supplementary material for: Super‐enhancer‐driven KIAA1522 upregulation suppresses ferroptosis in hepatocellular carcinoma
Source: Clin Transl Med. 2026 Jun 11;16(6):e70710. doi: 10.1002/ctm2.70710 (PMC13260693; doi:10.1002/ctm2.70710)
Supplement: Supplementary file 1 — Supporting Information [file CTM2-16-e70710-s001.docx]

**SUPPORTING INFORMATION**


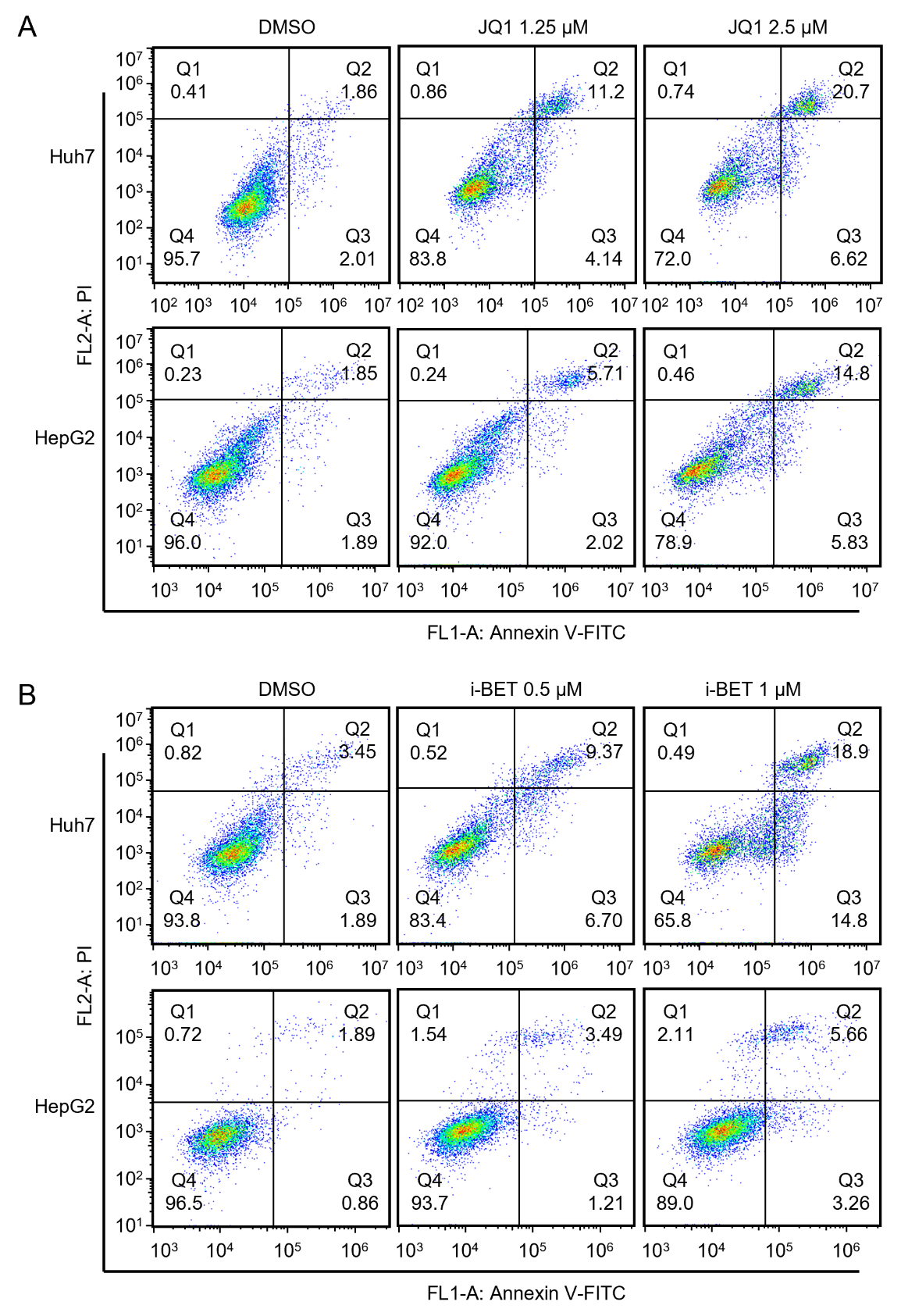


**FIGURE S1** Targeting SEs inhibits HCC cell proliferation. (A-B) The apoptosis rate of Huh7 and HepG2 cells treated with JQ-1 (1.25 μM and 2.5 μM) or i-BET151 (0.5 μM and 1.0 μM) for 48 h was determined by flow cytometry.


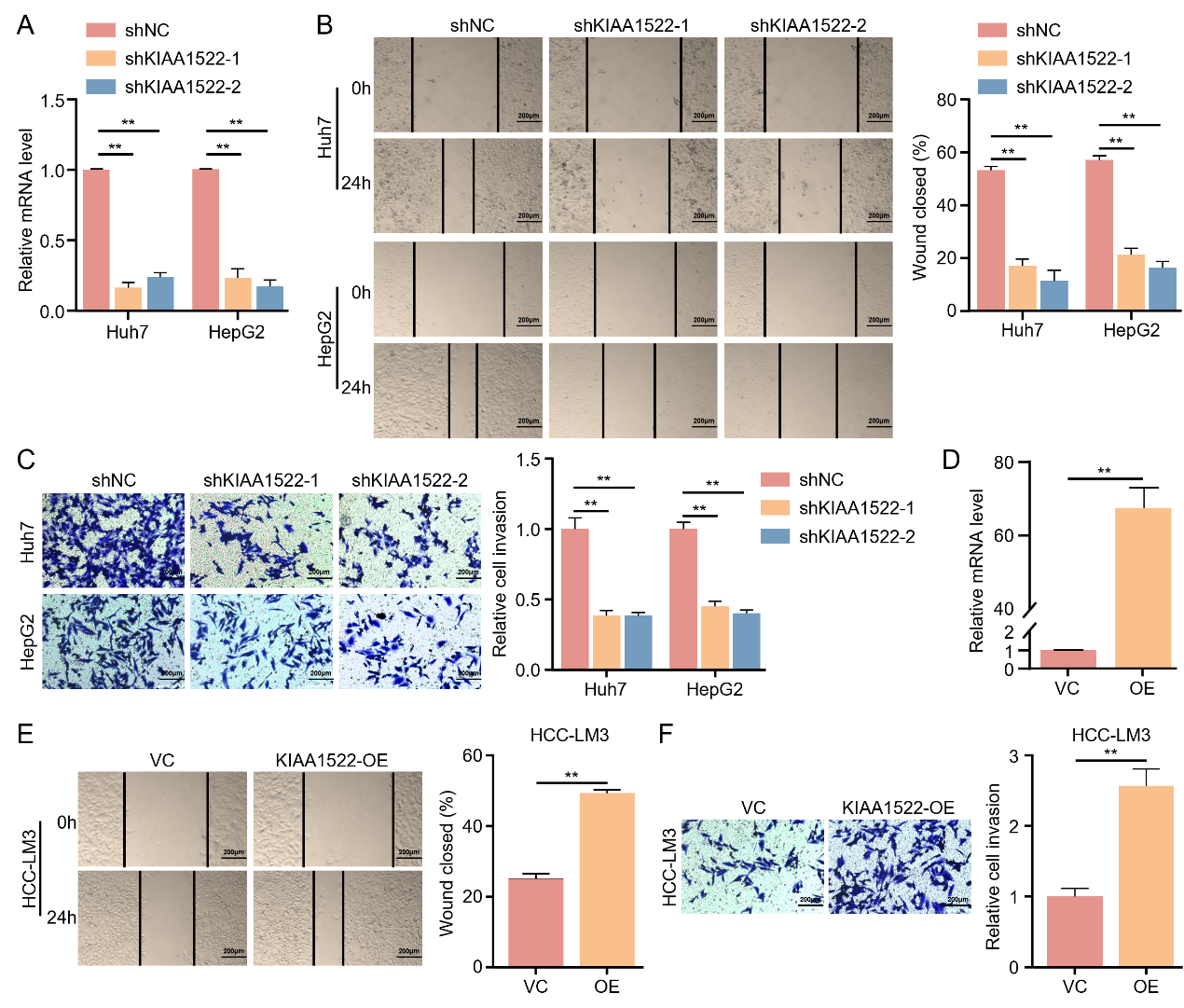


**FIGURE S2** KIAA1522 knockdown suppresses HCC cell migration and invasion. (A) Changes in KIAA1522 mRNA levels in Huh7 and HepG2 cells following KIAA1522 knockdown were detected by qRT-PCR. (B-C) Cell migration and invasion in Huh7 and HepG2 cells following KIAA1522 knockdown were measured using wound healing and Transwell assays. Scale bar: 200 μm. (D) Changes in KIAA1522 mRNA levels in HCC-LM3 cells following KIAA1522 overexpression were detected by qRT-PCR. (E-F) Cell migration and invasion in HCC-LM3 cells following KIAA1522 overexpression were measured using wound healing assay and Transwell assays. Scale bar:200 μm. Data represent the mean ± SD; ***p* < 0.01.


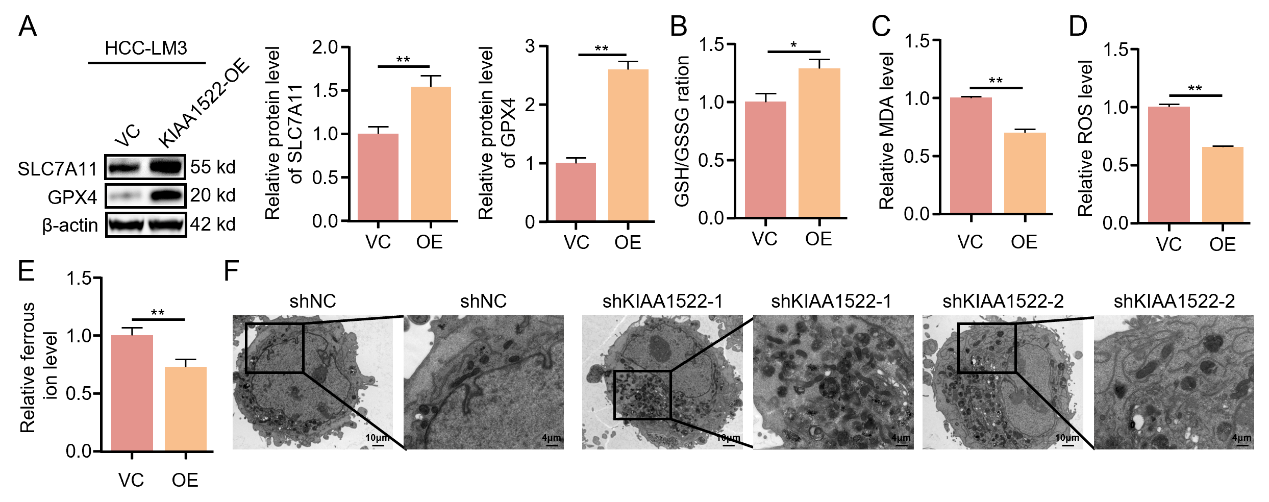


**FIGURE S3** KIAA1522 overexpression promotes resistance to ferroptosis in HCC. (A) The protein levels of SLC7A11and GPX4 in HCC-LM3 cells following KIAA1522 overexpression were detected by western blotting. (B) The GSH/GSSG ratio in HCC-LM3 cells following KIAA1522 overexpression was determined using the GSH/GSSG assay kit. (C) The MDA levels in HCC-LM3 cells following KIAA1522 overexpression were determined using the MDA assay kit. (D) The ROS levels in HCC-LM3 cells following KIAA1522 overexpression were determined by flow cytometry. (E) The ferrous iron levels in HCC-LM3 cells following KIAA1522 overexpression were determined using the ferrous ion assay kit. (F) Representative electron micrographs in Huh7 cells following shNC or KIAA1522 knockdown (shKIAA1522-1 and shKIAA1522-2) intervention were captured using transmission electron microscopy. Narrowed view, scale bar: 10 μm; enlarged view, scale bar: 4 μm. Data represent the mean ± SD; **p* < 0.05, ***p* < 0.01.


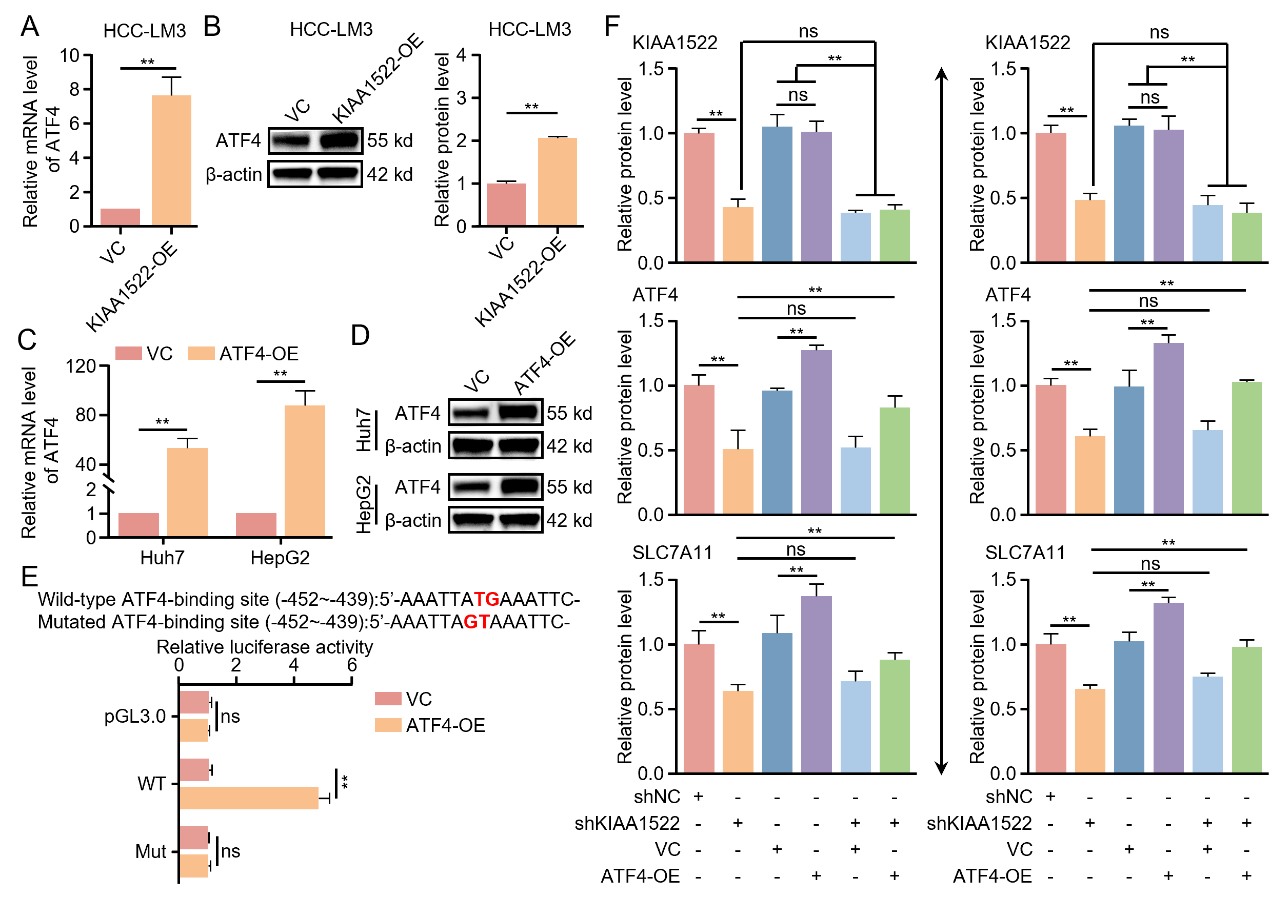


**FIGURE S4** KIAA1522 knockdown activates ferroptosis through ATF4. (A-B) The mRNA and protein levels of ATF4 in HCC-LM3 cells following KIAA1522 overexpression were analyzed by qRT-PCR and western blotting, respectively. (C-D) The mRNA and protein levels of ATF4 in Huh7 and HepG2 cells following ATF4 overexpression were analyzed by qRT-PCR and western blotting, respectively. (E) Relative luciferase activity in different groups was determined using a dual-luciferase reporter assay. (F) Statistical analysis of the protein levels of KIAA1522, SLC7A11, and ATF4 in Huh7 and HepG2 cells following KIAA1522 knockdown and/or ATF4 overexpression. Data represent the mean ± SD; ***p* < 0.01, ns, not significant.

**Table S1** The sequence of plasmids constructed in this study

| **Target** | **Strand** | **Sequence (5'-3')** |
| --- | --- | --- |
| shNC | Sense | CCTAAGGTTAAGTCGCCCTCG |
|  | Antisense | CGAGGGCGACTTAACCTTAGG |
| shKIAA1522-1 | Sense | CCTTGTAGCTAATCAATCA |
|  | Antisense | TGATTGATTAGCTACAAGG |
| shKIAA1522-2 | Sense | CCTACAACACCAAGAGAAA |
|  | Antisense | TTTCTCTTGGTGTTGTAGG |
| shBRD4-1 | Sense | GCTCAAGACACTATGGAAACA |
|  | Antisense | TGTTTCCATAGTGTCTTGAGC |
| shBRD4-2 | Sense | GCGTTTCCACGGTACCAAACA |
|  | Antisense | TGTTTGGTACCGTGGAAACG |
| sgRNA-P | Forward | AAGTCCCGCTCCGGCGCGTC |
|  | Reverse | GACGCGCCGGAGCGGGACTT |
| sgRNA-1 | Forward | GAGCTGACAGGCCGGTTCTA |
|  | Reverse | TAGAACCGGCCTGTCAGCTC |
| sgRNA-2 | Forward | GGGTCTGTGCGGTCGCTCAT |
|  | Reverse | ATGAGCGACCGCACAGACCC |
| KIAA1522-P1 | strand+ | hg38 chr1: 32741365–32741814 |
| KIAA1522-P2 | strand+ | hg38 chr1: 32741815–32742264 |
| KIAA1522-P3 | strand+ | hg38 chr1: 32742265–32742714 |
| Control-luc | strand+ | hg38 chr1: 32811310–32812947 |
| SE-luc | strand+ | hg38 chr1:32759454-32761091 |

**Table S2** The sequence of plasmids constructed in this study

| **Name** | **Sequence (5'→3')** |
| --- | --- |
| KIAA1522-F (homo) | TGGATGAGCACCAGGACAAC |
| KIAA1522-R (homo) | GTCCGGGAGGACTGGATACT |
| BRD4-F (homo) | CGTCAAGCTGAACCTCCCTG |
| BRD4-R (homo) | TGTCATCTCCAGGCTTGTTGTA |
| ATF4-F (homo) | CGCAACATGACCGAAATGA |
| ATF4-R (homo) | TCTCCAGCGACAAGGCTAAG |
| β-actin-F (homo） | GGCTGTATTCCCCTCCATCG |
| β-actin-R (homo） | CCAGTTGGTAACAATGCCATGT |
| BRD4-E1 Chip F-primer (chr1:32759454-32760182) | CCCAACTGCCCTTACCCATT |
| BRD4-E1 Chip R-primer (chr1:32759454-32760182) | CCAGGAGGCTCAAGGGAAAG |
| BRD4-E2 Chip F-primer (chr1:32760364-32761091) | GTGTCACTCTGTCACCCAGG |
| BRD4-E2 Chip R-primer (chr1:32760364-32761091) | GTCGAGCTCCTGTAATCCCG |
| BRD4-E3 Chip F-primer  (chr1: 32741365-32742725) | CTGGGTCTGGAAGGTCAACC |
| BRD4-E3 Chip R-primer  (chr1: 32741365-32742725) | CACTGTGCTCCATGTGAGGT |

**Table S3** Antibodies used in this study

| **Antigen_name** | **Host** | **Vendor** | **Catalogue** | **Dilution (WB)** |
| --- | --- | --- | --- | --- |
| KIAA1522 | Rabit | HUABIO | ER1911-85 | 1:500 |
| SLC7A11 | Rabit | Abcam | ab175186 | 1:1000 |
| GPX4 | Rabit | Abcam | ab125066 | 1:1000 |
| FTH1 | Rabit | Thermo Scientific | 701934 | 1:500 |
| ATF4 | Rabit | HUABIO | ET1612-37 | 1:1000 |
| Bcl-2 | Mouse | Cell signaling | 15071T | 1:1000 |
| Bax | Rabit | HUABIO | ET1603-34 | 1:1000 |
| β-actin | Rabit | Abmart | P60035M | 1:1000 |
| Goat Anti-Mouse IgG HRP | Goat | Abmart | M21001 | 1:3000 |
| Goat Anti-Rabbit IgG-HRP | Goat | Abmart | M21002 | 1:3000 |
